# IGF1R upregulation confers resistance to isoform-specific inhibitors of PI3K in *PIK3CA*-driven ovarian cancer

**DOI:** 10.1038/s41419-018-1025-8

**Published:** 2018-09-20

**Authors:** Jonatan Zorea, Manu Prasad, Limor Cohen, Nan Li, Roman Schefzik, Susmita Ghosh, Barak Rotblat, Benedikt Brors, Moshe Elkabets

**Affiliations:** 1 The Shraga Segal Dept. of Microbiology, Immunology and Genetics, Faculty of Health Sciences, Ben-Gurion University of the Negev, Beer-Sheva, 84105 Israel; 2 Division of Somatic Evolution and Early Detection, German Cancer Research Center (DKFZ), Heidelberg, Germany; 3 Faculty of Biosciences, Heidelberg University, Heidelberg, Germany; 4 Department of Life Sciences and the National Institute for Biotechnology in the Negev, Ben-Gurion University of the Negev, Beer-Sheva, Israel; 5 Division of Applied Bioinformatics, German Cancer Research Center (DKFZ), Heidelberg, Germany

## Abstract

Genomic alterations (GA) in *PIK3CA* leads to the hyper-activation of the phosphatidylinositol-4, 5-bisphosphate 3-kinase (PI3K) pathway in more than 20% of ovarian cancer (OC) patients. Therefore, PI3K therapies are under clinical evaluation for this subset of patients. Evidently, in clinical trials testing the efficacy of isoform-specific inhibitors of PI3K (PI3Ki), patients having a stable disease eventually relapse, as tumors become resistant to treatment. Hence, there is an urgent clinical need to develop new therapeutic combinations to improve the efficacy of PI3Ki in PIK3CA-driven OC patients. Here we identified the molecular mechanism that limits the efficacy of the beta-sparing PI3Ki, Taselisib (GDC0032), in *PIK3CA*-mutated OC cell lines (IGROV1 and OAW42) that acquired resistance to GDC0032. By comparing the molecular profile of GDC0032-sensitve and -resistant OC cell lines, we found that AKT/mTOR inhibition is required for GDC0032 efficacy. In resistant cells, the sustained activation of AKT/mTOR was regulated by the upregulation of the insulin growth factor 1 receptor (IGF1R). Knockdown of IGF1R re-sensitized cells to GDC0032 in vitro, and the combination of AEW541, an IGF1R inhibitor, with GDC0032 exhibited potent anti-tumor activity in vitro and in vivo. We further demonstrated that IGF1R regulates tumor cell proliferation in IGROV1 cells, whereas in OAW42, it determines autophagy as well. Overall, our findings suggest that the dual inhibition of PI3K and IGF1R may be considered as a new therapeutic strategy in *PIK3CA*-driven OC.

## Introduction

The phosphatidylinositol 3-kinase (PI3K) pathway is a key regulator of the survival, growth, and metabolism of normal and malignant cells^1,2^. This pathway is often hyperactivated due to genomic alterations (GA) in PI3K pathway-related genes such as deletions in *PTEN*, and mutations or amplifications in *PIK3CA, AKT3, AKT2, and AKT1*^3–7^. In ovarian cancer (OC) patients, point mutations or amplifications of the *PIK3CA* gene, which encodes the p110α catalytic subunit of the PI3K complex, are present in >20% of all OC types^3–6^. Around 240,000 OC patients are diagnosed annually; of these, ~50,000 harbor GA in *PIK3CA*^8^. Therefore, identifying potent anti-cancer therapies for this subset of OC patients is essential.

Cancer cells with GA in *PIK3CA* have been shown to be more susceptible to isoform-specific inhibitors of the PI3K pathway (PI3Ki) in vitro, in vivo, and in patients than tumor  cells with wild-type *PIK3CA*^9–19^. One of the most promising PI3Ki is GDC0032, a small molecule that blocks p110α, p110δ, and p110γ (p110β sparing). GDC0032 has exhibited clinical activity in tumors harboring *PIK3CA* alterations in early clinical trials^14,15^, but eventually all patients relapsed and developed resistance. Such results suggest that treating *PIK3CA*-driven OC with PI3Ki is a good therapeutic strategy, but new therapeutic approaches, such as drug combinations are needed to improve and prolong the positive clinical response.

Currently, two-thirds of the ongoing clinical trials testing PI3K-related therapies focus on combining at least two modalities^20^. In OC, ongoing clinical trials have been testing combinatorial PI3K therapies only with chemotherapy (NCT02069158 and NCT02476955). Hence, a better understanding of the molecular response and resistance to PI3Ki can lead to investigating more potent drug combinations in this malignant disease.

Our present study sought to identify the molecular mechanism that limits the efficacy of GDC0032, in *PIK3CA*-driven OC, to identify new therapeutic targets, and to examine new drug combinations.

## Results

### Sustained activation of the AKT/mTOR pathway is associated with resistance to GDC0032 in *PIK3CA*-mutated OC cell lines

**Fig. 1:**
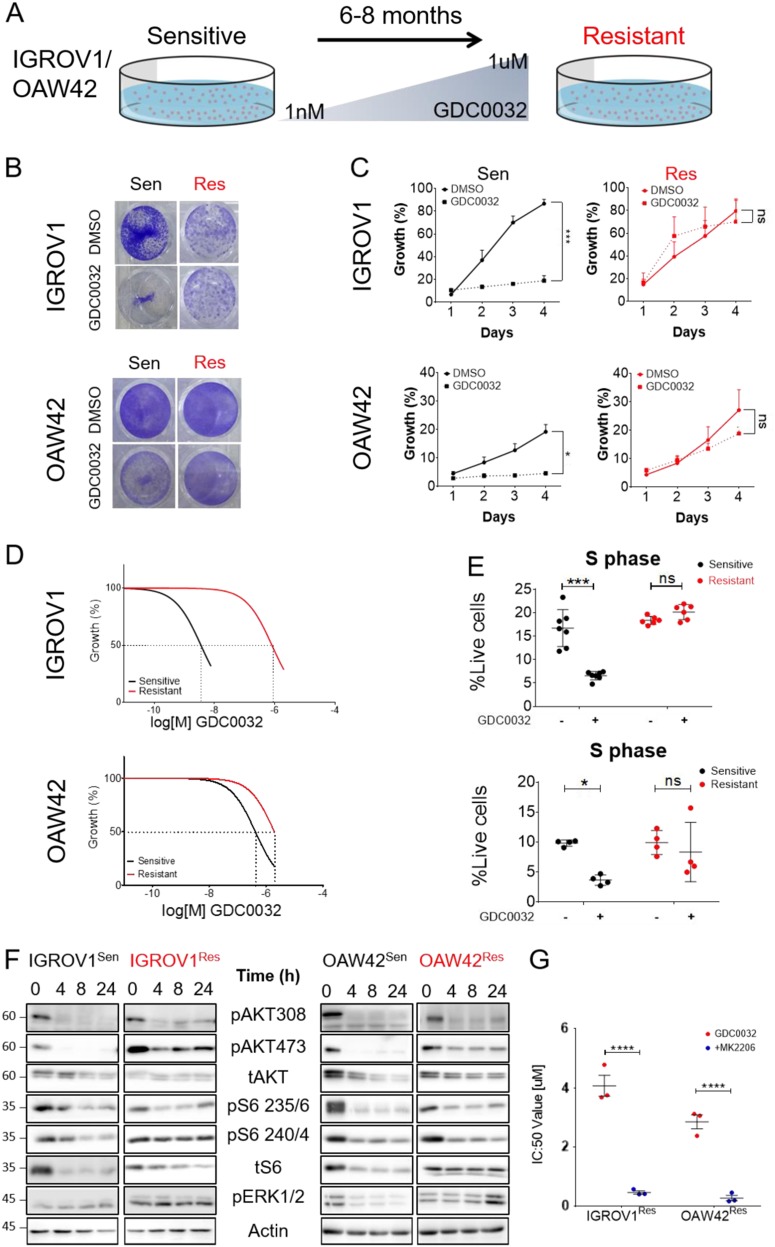
Characterizing the phenotype of the GDC0032-acquired -resistant cell lines. **A** A scheme illustrating the transformation of sensitive cells to resistant by exposing cells to increasing concentrations of GDC0032, starting at 1 nM and increasing up to 1 uM (see Material & Methods section). **B**, **C** Proliferation of IGROV1 and OAW42 sensitive (black) and resistant (red) cells, treated with DMSO or 100 nM GDC0032 for 4 days. The graphs (**C**) Scatter plot represents the growth, monitored by a live cell imager every 24 h (mean ± S.E.M. *n* = 3, *P < 0.05, ***P < 0.001). After 4 days of incubation, cells were fixed and stained using crystal violet (**B**). **D** GDC0032 IC:50 values of IGROV1^Sen^ (3.5e-9M) and IGROV1^Res^ (8e-7M) or OAW42^Sen^ (4.2e-7M) and OAW42^Res^ cells (1.9e-6M). (*n* = 6) **e** FACS analysis of the population in S phase in all cell lines, 48 h after treatment with DMSO or 100 nM GDC0032 (mean ± S.E.M. *n* > = 4, *P < 0.05, ***P < 0.001). **F** Whole-cell lysate western blot analysis of AKT, S6, and ERK from both cell lines, sensitive and resistant, in a time series of 100 nM GDC0032 treatment, compared to the beta-actin level. **G** GDC0032 IC:50 values of IGROV1^Res^ and OAW42^Res^ with the AKT inhibitor, MK2206 compared to GDC0032 alone showed in a scatter plot (mean ± S.E.M. *n* = 3, ****P < 0.0001)

Since *PIK3CA*-mutated cancer cells were found to be more sensitive to PI3Ki than were PIK3CA-wild-type cells^9–18^, we explored the Cancer Cell Line Encyclopedia (CCLE) databases^21^, searching for PI3Ki-sensitive OC cell lines with GA in *PIK3CA* (Supplementary Fig. 1A). We confirmed the sensitivity of two *PIK3CA*-mutated cell lines, IGROV1 and OAW42, to multiple PI3Kis, including the beta-sparing GDC0032 (Supplementary Fig. 1B). We noted that both cell lines exhibited the highest sensitivity to GDC0032. On the basis of the potency of GDC0032 in our cell lines and the clinical relevance of this agent, we decided to investigate how resistance to GDC0032 develops. To this end, we exposed these two tumor cell lines to increasing concentrations of GDC0032 for 6 to 8 months until resistance had developed (Fig. 1A). To verify the resistance phenotype, we checked their sensitivity to the same list of PI3Ki and noticed a reduction in the sensitivity to all inhibitors, emphasizing that resistance is not drug specific (Supplementary Fig. 1B). These acquired-resistant cell lines, IGROV1^**Res**istant^ and OAW42^**Res**istant^, displayed less growth inhibition in response to GDC0032 compared with parental cells, IGROV1^**Sen**ensitive^ and OAW42 ^**Sen**sitive^ (Fig. 1B, C). These cells also displayed a significantly higher half-maximal inhibitory concentration (IC:50) of GDC0032 compared with their corresponding IGROV1^Sen^ and OAW42^Sen^ cell lines (*p* value < 0.01) (Fig. 1D). Lastly, cell cycle analysis showed that treating IGROV1^Sen^ and OAW42^Sen^ with GDC0032 induced S phase arrest, whereas no S phase inhibition was observed in the IGROV1^Res^ and OAW42^Res^ cell lines (Fig. 1E).

Given recent evidence that activation of the mTOR and MAPK pathways drives resistance to PI3Ki^9,10,18,22–25^, we measured the activity of the AKT/mTOR and MAPK signaling pathways in sensitive and resistant cell lines, in response to GDC0032. We analyzed the phosphorylation (activation) of AKT (pAKT_308_, pAKT_473_), mTOR (pS6_235/236,_ pS6_240/244_), and MAPK (pERK_42/44_) over time. The pAKT and pS6 levels revealed that GDC0032 inhibits the AKT/mTOR pathway in sensitive cell lines, whereas sustained activation was observed in both resistant cell lines (Fig. 1F). To clarify the role of AKT activation in resistance to GDC0032, we investigated the effect of the AKT inhibitor, MK2206, in re-sensitizing IGROV1^Res^ and OAW42^Res^ cells to GDC0032. Cell proliferation assays showed that AKT inhibition enhances sensitivity to GDC0032 (Fig. 1G), proving that blockage of the AKT pathway is required for maintaining the anti-tumor potency of GDC0032.

### IGF1R expression confers resistance to GDC0032 in *PIK3CA*-mutated OC cell lines

**Fig. 2:**
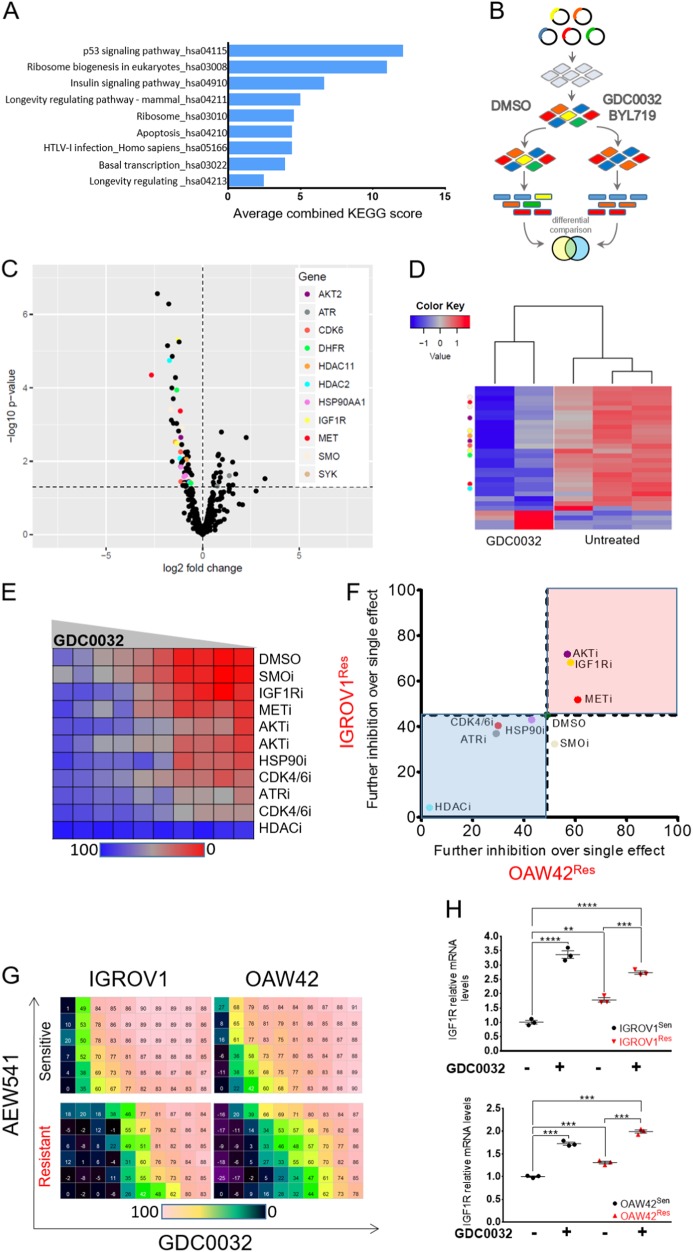
IGF1R confers resistance to GDC0032. **A** Bar graph showing the nine shared enriched upregulated pathways, scored by averaging the KEGG combined scores for each pathway of each cell line. **B** A scheme illustrating the synthetic lethality shRNA screen workflow, done on IGROV1^Res^ cells. **C** Volcano plot visualizing the results (log2 fold change against –log10 *p* value) for the comparison between those cells treated with GDC0032 and the control cells. Each dot corresponds to one shRNA. Genes with at least two shRNAs with significant differential expression that were all consistently either up- or downregulated are denoted in color (see the color index). **D** Heatmap across all samples displaying the top 30 differentially expressed shRNAs. Colors on the right indicate the genes, and match the color index of the former panel (2 C). **E** A drug matrix showing the sensitivity of IGROV1^Res^ cells to multiple inhibitors, combined with increasing concentrations of GDC0032. **F** The inhibitory effect of each drug was calculated for both IGROV1^Res^ and OAW42^Res^ in the presence of 0.5 uM of GDC0032. Inhibitors that had a higher effect than GDC0032 alone for both cell lines are in the red quartile. **G** A dose matrix used to calculate the synergistic effect of combining GDC0032 and AEW541. The concentration of AEW541 increases from the bottom to the top and the concentration of GDC0032 from the left to the right. **H** Scatter plot showing the mRNA levels of *IGF1R* before and after GDC0032 treatment for both IGROV1 and OAW42, sensitive (black) and resistant (red) cells. (mean ± S.E.M. *n* = 3, **P < 0.01, ***P < 0.01, ****P < 0.0001)

To identify specific candidates mediating resistance to GDC0032, we compared the mRNA profile of the resistant and the sensitive cells in each cell line. The mRNAs that were significantly upregulated in resistant cells (IGROV1^Res^ and OAW42^Res^), compared with their sensitive counterpart cells, were used to create gene set enrichment lists. We then looked for pathways that were upregulated in both cell lines and calculated the average combined KEGG score for each. We found nine shared pathways, including P53 and the insulin signaling pathway (Fig. 2A). In addition, we performed a targeted synthetic lethality shRNA screen using IGROV1^Res^ cells (Fig. 2B). Briefly, we infected IGROV1^Res^ cells with a library of ~600 shRNAs, knocking down ~132 targetable genes as previously described^9,10^. Next, we treated the infected cells with vehicle (DMSO) or GDC0032 for 14 days, and analyzed the number of all shRNA under each condition. The analysis revealed that the proliferation of IGROV1^Res^ cells, infected with shRNAs targeting *IGF1R, MET, AKT*, and others, were strongly inhibited in GDC0032 compared with DMSO-treated cells (Fig. 2C, D, also performed with BYL719- Supplementary Fig. 2A). Taken together, both methods indicated that the insulin signaling pathway, and specifically IGF1R, may play a role in acquiring resistance to GDC0032.

We further validated the role of IGF1R and other candidates observed in our shRNA screening results by using pharmacological inhibitors. We measured the proliferation of the IGROV1^Res^ and OAW42^Res^ tumor cell lines treated with increasing concentrations of GDC0032, in the absence or presence of inhibitors such as AEW541 (IGF1R inhibitor), PHA-665752 (MET inhibitor), MK2206 (AKT inhibitor), and others (Supplementary Fig. 2B). We noted that the inhibition of IGF1R, MET, and AKT had a greater anti-tumor effect when combined with GDC0032 in both IGROV1^Res^ and OAW42^Res^ (Fig. 2E, F, and Supplementary Fig. 2C).

Since MET and IGF1R expression limited the efficacy of GDC0032, we investigated the effect of these receptors on tumor cell viability in the presence of GDC0032 by conducting a head-to-head synergistic drug combination^26^. We tested the proliferation of cells treated with single agents IGF1Ri (AEW541), METi (PHA-665752), and GDC0032 compared to their combinations. Importantly, we found that combining GDC0032 with AEW541 has a potent synergistic anti-proliferative effect, whereas combining GDC0032 with PHA-665752 displayed an additive effect (Fig. 2G and Supplementary Fig. 2D). Similar synergistic anti-tumor activity was also confirmed with BYL719 (Supplementary Fig. 2D). This synergy between these agents highlights the importance of blocking IGF1R for better efficacy of GDC0032 and BYL719.

Owing to the direct role of IGF1R in sensitivity to GDC0032, and the upregulation of the insulin signaling pathway in resistant cells, we quantified its mRNA levels in sensitive and resistant cells before and after treatment with GDC0032. Quantitative PCR (qPCR) analysis revealed that the basal expression levels of IGF1R are higher in resistant cells compared with their counterpart sensitive cells. Furthermore, treatment of cells with GDC0032 increases the expression of IGF1R in both tumor cell line models (Fig. 2H). This direct effect of GDC0032 treatment on IGF1R expression, together with our functional re-sensitization studies, encouraged us to further explore how inhibiting IGF1R, using AEW541, is involved in re-sensitizing cells to GDC0032 in vitro and in vivo.

### Dual treatment with AEW541 and GDC0032 enhances tumor growth arrest in vitro and in vivo, via potent inhibition of the AKT/mTOR pathway

**Fig. 3:**
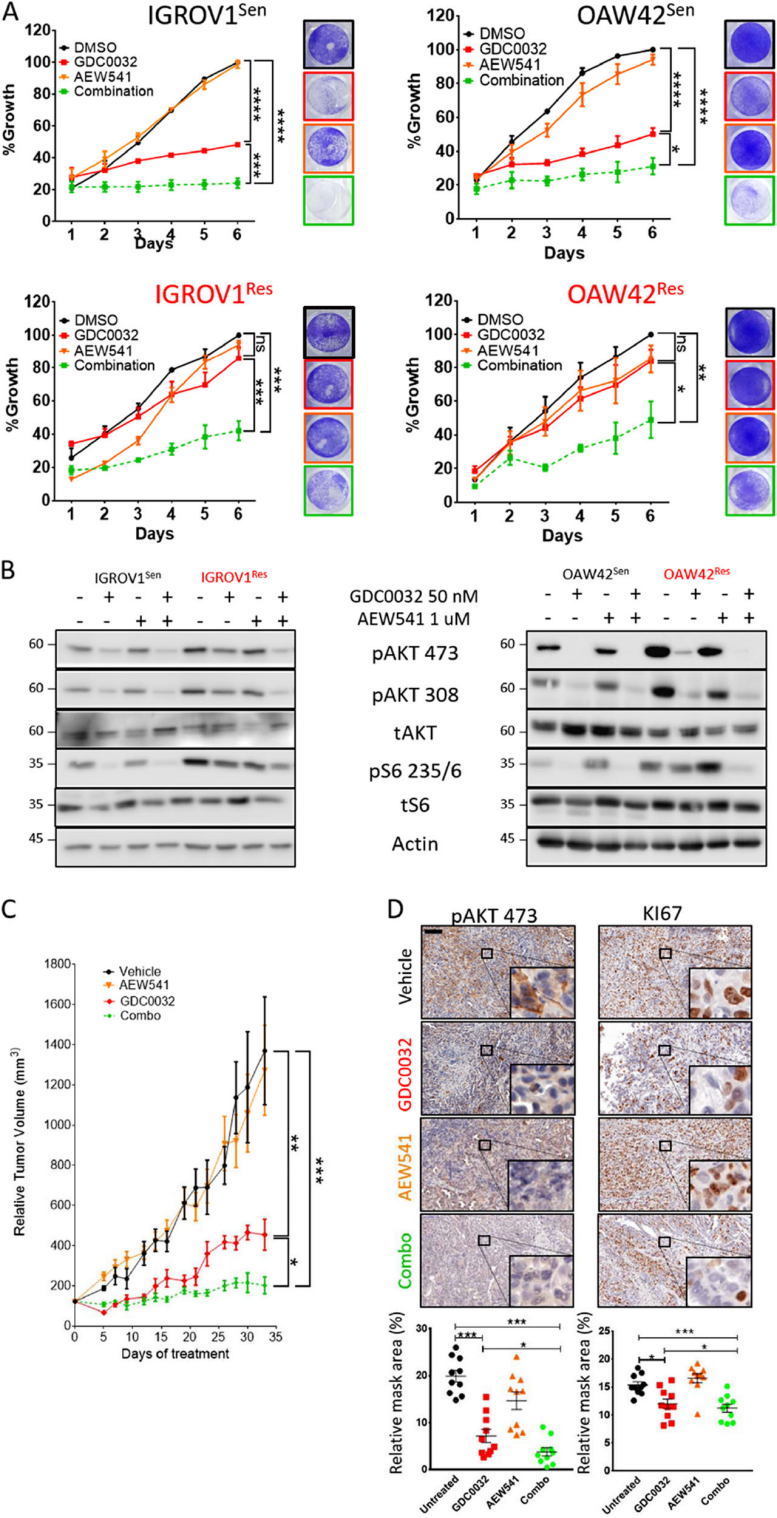
Combining GDC0032 and AEW541 enhances tumor growth arrest. **A** The growth of IGROV1 and OAW42, sensitive and resistant cells, treated with DMSO (black), GDC0032 (red), AEW541 (orange), or AEW541-GDC0032 (green) was monitored by a live cell imager every 24 h and quantified by JuliStat. After 6 days of incubation, cells were fixed and stained using crystal violet (mean ± S.E.M. *n* = 3, *P < 0.05, **P < 0.01, ***P < 0.01, ****P < 0.0001). **B** Whole-cell lysate western blot analysis of AKT, and S6 from both cell lines, sensitive and resistant, treated for 24 h as indicated. **C** Four arms of tumor-bearing mice were treated daily as indicated. Tumor dimensions were measured every 2 days using a caliper and the relative tumor volume was calculated using the formula *V* = (*L* × *W* × *W*) / 2, where *V* is the tumor volume, *W* is the tumor width, and *L* is the tumor length. (mean ± S.E.M. *n* (of mice in a group) > = 4, *P < 0.05, **P < 0.01, ***P < 0.01). **D** KI67 and pAKT473 IHC staining of tumors from the four arms as indicated (scale bar of 100um). The relative mask area of each arm was calculated and presented in a bar graph below each image panel. (mean ± S.E.M. *n* = 10, **P < 0.01, ***P < 0.01, ****P < 0.0001)

We further investigated the biological and molecular response to single and combined treatment with GDC0032 and AEW541, with both sensitive and resistant cells. An imaging-based proliferation assay showed that combined treatment with AEW541 and GDC0032 further reduced cancer cell proliferation in sensitive and resistant cell lines compared with the single drug inhibition treatment (Fig. 3A). Western blot analysis of the signaling pathway indicated that this combined treatment with GDC0032 and AEW541 further inhibits the AKT/mTOR pathway, compared with single agents (Fig. 3B).

To test the efficacy of combining GDC0032 and AEW541 in vivo, we created a cell line-derived xenograft model by implanting IGROV1^Sen^ cells (OAW42 tumor cells were not tumorigenic) into NOD/SCID mice and treating them with vehicle, GDC0032 (10 mg/kg per day) and AEW541 (30 mg/kg per day), separately and combined. As expected, GDC0032 induced a growth delay of IGROV1^Sen^ tumors, since in the first 2 weeks no tumor progression was detected. Similar to the observation in patients, the tumors eventually started to grow (after 2 weeks of treatment), and tumor volumes were increased by 4-fold, despite daily treatment using GDC0032. Treatment with AEW541 had no effect on tumor growth, compared with the vehicle-treated group. However, combining both drugs (GDC0032 and AEW541) resulted in superior anti-tumor activity, with no appreciable increase in tumor volume, even following 30 days of treatment (Fig. 3C). In agreement with this, histopathological analysis of the tumor tissue showed that co-treatment with GDC0032 and AEW541 reduces tumor cell proliferation, as measured by KI67, and further inhibits the AKT pathway (Fig. 3D). No indication for increased cell death was detected by TUNEL staining (Sup Fig. 3C). Two additional treatments were examined in this experiment: BYL719 alone and combined with AEW541. As shown in Supplementary Figure 3A and B, supplementation of AEW541 enhances tumor sensitivity to BYL719. Overall, these results indicate that blocking IGF1R with AEW541 enhances the efficacy of GDC0032 and BYL719 in vitro and in vivo, by preventing AKT/mTOR pathway activation.

### IGF1R signaling determines tumor cell proliferation and autophagy

**Fig. 4:**
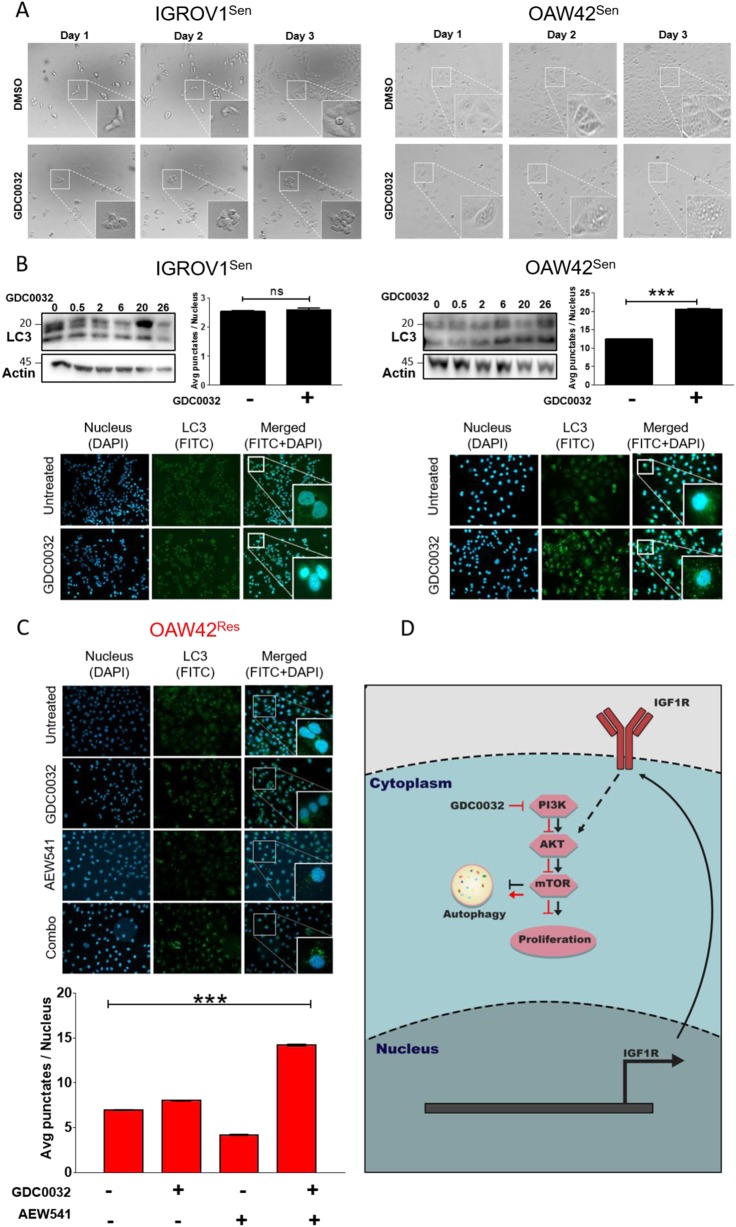
IGF1R determines autophagy in OAW42 cells. **A** Live cell imaging showing the formation of punctate structures in OAW42^Sen^ cells treated with GDC0032 over time. **B** The changes in LC3B levels in both OAW42^Sen^ and IGROV1^Sen^ cells were first quantified by whole-cell lysate western blot analysis in a time series (hours) of 50 nM GDC0032 treatment. Second, punctate structures were stained after 24 h of treatment, using the Cyto-id kit and the average number of punctate structures per nucleus was calculated using the ImageStream count feature (mean ± S.E.M. *n* > 1000, ***P < 0.01). Last, punctate structures were imaged using a Zeiss inverted microscope (×40). **C** OAW42^Res^ cells treated as indicated and the average number of punctate structures per nucleus was imaged using an Zeiss inverted microscope (X40) and calculated using the ImageStream count feature. (mean ± S.E.M. *n* > 1000, ***P < 0.01). **D** A scheme summarizing the resistance mechanism involving IGF1R in OAW42^Res^ cells

To further explore the role of IGF1R in the tumor cells’ response to GDC0032, we examined the morphology of tumor cells after treatment with GDC0032 over time. In IGROV1^Sen^ cells, GDC0032 induced potent tumor growth arrest, with no evidence of cell death (floating cells) or autophagy (vesicles in the cells). In contrast, in OAW42^Sen^ cells, we observed vesicle formation following GDC0032 treatment, together with reduced cell proliferation (Fig. 4A). Given recent evidence showing the role of autophagy in resistance to anti-cancer therapies, including PI3K/AKT therapies^27,28^, and the involvement of IGF1R in autophagy^29,30^, we investigated the role of IGF1R in GDC0032-induced autophagy in our OC cells. Specifically, we measured the LC3B levels (western blots) and autophagosome accumulation (using a florescence-based dye), following GDC0032 treatment. Analysis of IGROV1^Sen^ and OAW42^Sen^ tumor cells revealed that treatment with GDC0032 induces the accumulation of LC3B and the appearance of autophagosomes only in OAW42^Sen^ tumor cells (Fig. 4B; Supplementary Fig. 4). In contrast to OAW42^Sen^ cells, in OAW42^Res^ cells GDC0032 did not induce autophagy. However, combined treatment using AEW541 and GDC0032 induced the autophagy phenotype in OAW42^Res^ cells, indicated by autophagosome formation (Fig. C4).

Overall, we showed that blocking the AKT/mTOR pathway is required for the efficacy of GDC0032 (and BYL719), in *PIK3CA*-mutated OC cells. Resistance to GDC0032 develops via the upregulating of IGF1R, which reactivates the AKT/mTOR pathways. OAW42 undergoes autophagy after GDC0032 treatment to avoid cell death, and upregulation of IGF1R enables cells to escape from autophagy and to propagate (Fig. 4D). Blocking IGF1R sensitizes OC cells to GDC0032 (and BYL719) in vitro and in vivo; thus, combining these drugs may be considered for OC patients with GA in *PIK3CA.*

## Discussion

Resistance to PI3K inhibitors is a major obstacle in developing these agents in oncology. The molecular mechanisms that induce resistance to PI3K therapies vary from epigenetic regulators, autocrine, or paracrine secretion of pro-survival factors, to overexpression or activation of RTK^20^. In our acquired-resistant model, we found that overexpression of IGF1R plays a major role in maintaining AKT/mTOR pathway activation. Inhibition of IGF1R enhances the sensitivity of *PIK3CA*-driven OC to GDC0032 and BYL719 treatment in vitro and in vivo.

IGF1R is frequently overexpressed in tumors such as thyroid, colorectal, prostate, breast, and ovarian, and regulates tumor cell proliferation and survival via apoptotic signaling^31^. The signaling of IGF1R plays a role in tumor cell motility and adhesion; thus, it contributes to tumor spreading and metastasis^32^. IGF1R is considered an attractive target in oncology;^33^ however, years of clinical research yielded only modest efficacy in cancer patients treated with a single agent of anti-IGF1R therapies^34,35^. These clinical results highlight the insignificant role of IGF1R as a driver oncogene. Owing to its important role in cancer progression and in limiting the efficacy of anti-cancer therapies, blocking IGF1R combined with other inhibitors is under clinical development in various cancer types, including melanoma^36^, lung^37^, breast^38^, and prostate cancers^39^. In OC, IGF1R is known to confer resistance to cisplatin, paclitaxel, and cisplatin-taxol^40^, and to BEZ235 inhibitor as well^41^. Here we show the first evidence that IGF1R triggers resistance to isoform specific inhibitor of the PI3K in *PIK3CA*-driven OC.

Recently, several publications demonstrated the mechanistic role that IGF1R plays in promoting resistance to PI3K/AKT/mTOR inhibitors. In three separate studies, IGF1R was shown to re-activate AKT/mTOR through interaction with either p110β^25^, the integrin alpha2/FAK complex^42^, or pyruvate dehydrogenase kinase (PDK1)^43,44^, in various cancers. In our OC cell line models, p110β seems to play no role in resistance to GDC0032, since AZD6482, a p110β inhibitor, did not re-sensitize the acquired resistant cells to GDC0032 (Supplementary Fig. 2E). Next, regulation by interacting with the integrin alpha2/FAK complex seems highly unlikely, since there was no change in the PTK2 (*FAK1* gene) RNA expression (observed in IGROV1 cells, Supplementary Table 1). Lastly, preliminary results in our lab showed that blocking PDK1 together with GDC0032 reduced the proliferation of IGROV1^Res^ cells. Therefore, we suspect that IGF1R interacts with PDK1; however, further experimentation is required.

Autophagy is a well-known resistance mechanism used in anti-cancer therapies^45^. Tumor cells that undergo this process degrade unneeded components and recycle amino acids to synthesize essential proteins required for survival. mTORC1 plays a key role in regulating autophagy, since its inhibition dephosphorylates Atg13, ULK1, and ULK2, which take part in initiating autophagy^46,47^. In one of our sensitive tumor cell models (OAW42), treatment with GDC0032-induced autophagy, whereas in the OAW42-resistant cells, it did not. This phenomenon can be explained by our finding that upregulated IGF1R re-activates the AKT/mTOR pathway. Furthermore, we suspect that IGROV1 cells did not develop GDC0032-induced autophagy due to the interplay between P53 and autophagy^47^. For example, colon cancer cells with wild-type, but not mutated P53, were re-sensitized to chemotherapy by inhibition of autophagy^47,48^, emphasizing the importance of P53 in determining autophagy. OAW42 cells have a wild-type P53 and thus induce autophagy in response to GDC0032 treatment, whereas IGROV1 cells with mutated P53, do not^21^.

In summary, we provide here the first evidence that IGF1R and autophagy play a role in resistance to isoform specific inhibitors of the PI3K in OC. Since GDC0032, BYL719, and AEW541 are under clinical evaluation, our results warrant the clinical testing of the dual inhibition of IGF1R and PI3K for treating *PIK3CA*-mutated and amplified OC patients.

## Materials and Methods

### Cell Lines

OAW42 and IGROV1 cells were obtained and grown as previously described^9^. Both tumor cell lines were maintained at 37 °C in a humidified atmosphere at 5% CO_2_, in DMEM or RPMI-1640 medium, respectively, supplemented with 1% l-glutamine 200 mM, 100 units of penicillin, streptomycin 10% fetal bovine serum, and 200 nM antimicrobial cocktail (De-Plasma^TM^, TOKU-E) to avoid mycoplasma infection. Testing for mycoplasma was done every 6 months using Hy-Mycoplasma PCR kit (KI 5034I, Life Technologies).

### Induction of GDC0032-resistant cell lines

GDC0032- acquired resistant cell lines were derived from their original parental cell line by continuous exposure to GDC0032. Tumor cells were cultured initially with low concentration of GDC0032 (IC:20, 1–10 nM) for 2 months. The concentration of GDC0032 in the culture was increased gradually by 50–100 nM every 2 weeks. This development period was carried out for 6 to 8 months.

### Antibodies and reagents

The antibodies anti-pAKT Ser473 (#4060), anti-pAKT thr308 (#9275), anti-AKT (#4691), anti-pS6 S235/236 (#4858), anti-pS6 S240/244 (#5364), anti-S6, anti-pERK1/2 Thr202/Tyr204 (#9101), and anti-ERK1/2 (#4695) were from Cell Signaling Technology. Anti-LC3B was obtained from Sigma-Aldrich (L8918). KI67 was purchased from Vector laboratories (#VP K451). Anti-Actin was from MP Biomedicals (691001). Novartis Pharma AG (Basel, Switzerland) provided BYL719 and AEW541. LDE225, MK2206, AZD6482,AZD6738, GDC0032, PHA-665752, 17-AAG, LEE011, LY2835219, LBH589, ABT737 and Navitoclax were purchased from Selleckchem (Houston TX, USA). TUNEL- TREVIGEN, cat no-4815-30-K).

### Western blotting

Cells were washed with ice-cold PBS and scraped into ice-cold lysis buffer with phosphatase inhibitor cocktail (Stratech, B15001-BIT). Lysates were cleared by centrifugation at 14 000 rpm for 10 min at 4 °C, and supernatants were collected and assayed for protein quantification using the Bradford protein assay (Biorad, 5000006). Quantified samples were then analyzed by western blotting. Twenty micrograms of total lysate were resolved on NuPAGE 4–12% Bis-Tris gels and electrophoretically transferred to PVDF membranes (Biorad, 1704157). Membranes were blocked for 1 h in 5% BSA in Tris-buffered saline (TBS)-Tween and then hybridized using the primary antibodies in 5% BSA TBS-Tween. Mouse and rabbit horseradish peroxidase (HRP)-conjugated secondary antibodies (1:20 000, GE Healthcare) were diluted in 5% BSA in TBS-Tween. Protein-antibody complexes were detected by chemiluminescence with ECL λernova (Cyanagen) and images were captured with a c300 azure camera system. Protein bands were analyzed by comparing their size to SMOBIO-PM-2600 protein marker.

### Cell Viability and Synergy Assays

About 3500 cells were seeded in a 96-well plate. At 24 h after seeding, cells were treated with increasing concentrations of the indicated drug, left to right, and for the synergy assay with another drug, top to bottom. At 96 h post treatment, the medium was aspirated, and cells were fixed using Trichloroacetic acid (TCA) 0.6 M for 1 h at 4 °C. TCA was then washed, plates were dried on filter paper, and then incubated with 0.6% Crystal violet staining solution at room temperature on a bench rocker. After 10 min, plates were washed, air dried, and incubated with Acetic Acid (10%) for 10 min. The optical density of each well was measured at 570 nm using an Epoch Microplate Spectrophotometer.

For the synergy assay, the colorimetric results were analyzed using Chalice^TM^ Bioinformatics Software and the synergistic effects were calculated based on the statistic Loewe Excess model and the equation *S* = fcov ln fX ln fY ∑ max(0,Idata) max(0,Idata–ILoewe), which is a positive-gated, inhibition-weighted volume over Loewe additivity. Complete description of formula and calculation is available in http://chalice.horizondiscovery.com/chalice-portal/documentation/analyzer/analyticMethods.jsp.

### Xenograft Mouse Experiment

A total of 5 × 10^6^ IGROV-1 cells were suspended in 50 μl PBS and injected subcutaneously (S.C) into six NOD/SCID mice (female, 6–8 weeks old, ~20 g weight). When tumors reached a volume of 400 mm^3^, the tumors were dissected into 2 × 2 × 2 mm chunks and implanted S.C in 24 naive female NOD/SCID mice (6–8 weeks old). All 24 mice developed tumors and as tumor volume reached 150–200 mm^3^, mice were randomized into six groups of four mice (*n* = 8 tumors per group). Daily treatment of GDC0032 (10 mg/kg), BYL719 (25 mg/kg), AEW541 (30 mg/kg) or the combinations were given to mice by oral gavage.

Drugs were dissolved in 5% Carboxymethylcellulose (CMC). Vehicle-treated mice (control group) received 5% CMC. Tumors were measured 3 times a week by caliper and tumor volume was calculated using the formula: Volume = (Length × Width x Width) / 2. Mice were maintained and treated in accordance with the institutional guidelines of Ben-Gurion University of the Negev. Animal experiments were approved by the Institutional Animal Care and Use Committee (IL.80-12-2015). Mice were housed in air-filtered laminar flow cabinets with a 12 h light/dark cycle and food and water ad libitum. Tumor volumes are plotted as means ± SEM, and ANOVA test was applied.

### Immunohistochemistry

Immunohistochemistry (IHC) was performed using the ABC KIT (VECTASTAIN®ABC). After mice were euthanized, the dissected tumors were fixed in 4% formalin buffer overnight at room temperature, transferred to 70% ethanol, and embedded in paraffin, sectioned at 5 μm, loaded onto microscope slides, and deparaffinized at 60 °C for 1 h.

After the rehydration, antigen was retrieved. The slides were incubated in 10 mM citric acid buffer, pH 6.0 at 100 °C for 20 min, then cooled in buffer at room temperature for 20 min and rinsed with double distilled water for 3 min, 3 times. Next, endogenous peroxidases were inactivated by 0.3% hydrogen peroxide (H_2_O_2_) in Methanol buffer for 20 min; the buffer was washed 3 times with PBS for 3 min, and slides were blocked with 5% BSA in PBS-T for 1 h at room temperature. Primary antibodies were diluted in blocking solution and sections were incubated overnight at 4 °C with the antibodies. The next day, slides were washed with 1 × PBS with Tween three times, incubated with a specific biotinylated secondary antibody at room temperature for 1 h; the color developed by adding substrates. Last, slides were dehydrated and covered using mounting medium.

TUNEL assay was done according to manufacturer’s protocol (TREVIGEN, cat no-4815-30-K).

### Cell Cycle

For each condition, both cells and growth medium were collected into 15 ml tubes, centrifuged for 10 min at 4 °C, then a pellet was fixed using 70% ice-cold ethanol and stored for at least 24 h at −20 °C. After fixation, the pellet was washed twice with ice-cold 1X PBS, treated for 30 min at 37 °C with 100 μl of RNase solution (100 μg/ul), and stained in the dark for 20 min with 200ul Propidium iodide solution (100 μg/ml). Last, the cell phase was analyzed using BD FACSCANTO II.

### RNA sequencing

Cells were treated with 50 nM GDC0032 or DMSO for 24 h. RNA was extracted by using the PureLinkTM RNA mini kit (Invitrogen). After quality control of BioAnalyzer(RIN > 7), IGROV1 samples were sent to the g-INCPM institute at the Weizmann Institute of Science. Briefly, 500 ng of total RNA were fragmented, followed by reverse transcription and second strand cDNA synthesis. The DS-cDNA was subjected to end repair, a base addition, adapter ligation, and PCR amplification to create libraries. Libraries were evaluated by Qubit and TapeStation. Sequencing libraries were constructed with barcodes to allow multiplexing of 12 samples in one lane. A total of 17–20 million single-end 60-bp reads were sequenced per sample on an Illumina HiSeq 2500 V4 instrument.

### Imaging

Live cell imaging and time series recording were performed using the Juli^TM^ Stage Real-Time Cell History Recorder. Punctate structures were imaged using both the ImageStream and Zeiss Axio Observer 7. IHC slides were scanned using the Panoramic MIDI II scanner, 3DHISTECH.

### ShRNA screening

ShRNA screening was performed as described^49^. Briefly, IGROV1 cells were infected with pools of retroviral shRNA. 3 days post infection, cells were selected with puromycin for an additional 3 days (1 µg/ml) to remove the minority of uninfected cells. Thereafter, cells proliferated in culture for 3 days and then an initial population-doubling 0 (PD 0) sample was taken. The remaining population was divided into 9 groups: 3 groups kept as a control, 3 groups treated with GDC0032 (100 nM), and 3 groups treated with BYL719 (1 uM). Cells grew in the presence or absence of drugs for an additional 12 doublings before the final, PD 13 sample was taken. For the PD 0 and PD 13 samples, the shRNA HH barcode was PCR-recovered from genomic samples and samples were sequenced to calculate the abundance of the different shRNA probes. The change in the relative abundance of each shRNA in the library over time was measured using the normalized PD 13/PD 0 ratio of its reads. A log_2_(PD 13/PD 0) ratio of < 0 indicates that shRNA was depleted in the population over time, and a log_2_(PD 13/PD 0) ratio of ˃ 0 indicates that shRNA was enriched. To identify shRNAs that are synthetically lethal with GDC0032 or BYL719, the mean log_2_(PD 13/PD 0) ratios of the treated cell triplicates were compared to the control triplicates to derive the log_2_ ratio difference. A *p*-value of the difference between the two triplicates was calculated using the Student *t* test. Targets were filtered by the presence of at least two different shRNAs for the same gene and when *P* < 0.05.

### Statistical analysis

To analyze any differential expression in the IGROV1 cell lines, a generalized linear model likelihood ratio test was used. Specifically, a negative binomial generalized linear model was fitted, with shRNA-wise or gene-wise dispersions estimated using the Cox-Reid profile-adjusted likelihood method. Then, a likelihood ratio test was employed to check the differential expression^50^. The dispersion estimation, model fitting, and testing were implemented using the functions estimateDisp(), glmFit () and glmLRT(), respectively, in the edgeR package^51^

To interpret the testing results in a biological context, gene ontology (GO) enrichment analysis and KEGG pathway enrichment analysis were employed as downstream procedures, using the online available tool Enrichr^52^.

For pathological analysis, IHC images were analyzed by Histoquant software (3D Histech).

The remaining experiments were repeated at least three times and representative data/images are shown. Statistical analysis was performed using GraphPad Prism software, presented as mean ± SEM. For comparisons between two groups, P values were calculated. *P* values of 0.05 (*), 0.01 (**),0.001 (***) and 0.0001(****) were considered statistically significant.

## Electronic supplementary material

Supplementary Figures and legends

Supplementary Table 1
