# Supplementary material for: IGF1R upregulation confers resistance to isoform-specific inhibitors of PI3K in PIK3CA-driven ovarian cancer
Source: Cell Death Dis. 2018 Sep 20;9(10):944. doi: 10.1038/s41419-018-1025-8 (PMC6148236; doi:10.1038/s41419-018-1025-8)
Supplement: Supplementary file 1 — Supplementary Figures and legends [file 41419_2018_1025_MOESM1_ESM.pdf]

A

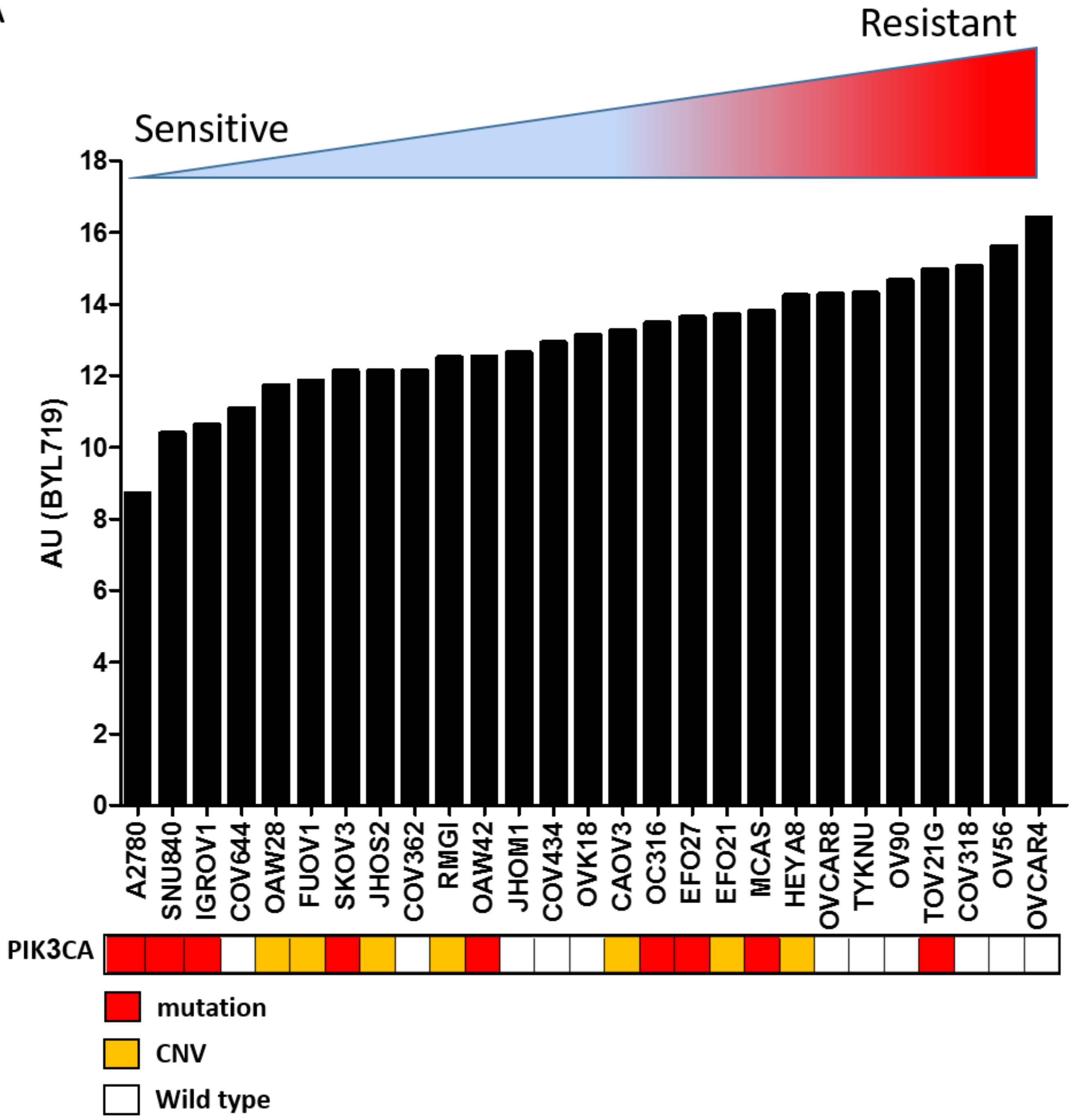

B

Inhibitor name	specificity	IC:50(M) for IGROV1 Sensitive	IC:50(M) for IGROV1 Resistant	IC:50(M) for OAW42 Sensitive	IC:50(M) for OAW42 Resistant
BKM120	p110α/β/δ/γ	7.95E-07	2.22E-06	4.14E-06	3.06E-06
PIK90	p110α/γ/δ	3.58E-07	4.21E-06	1.72E-06	5.06E-06
A66	p110α	1.28E-06	8.3E-06	3.06E-06	1.51E-05
XL147	p110α/δ/γ	4.85E-06	2.18E-05	2.66E-05	5.01E-05
BYL719	p110α	4.64E-07	2.74E-06	1.21E-06	4.69E-06
GDC0941	p110α/δ	4.94E-07	3.66E-06	4.26E-07	1.4E-06
GDC0032	p110α/δ/γ	1.59E-07	3.56E-06	2.34E-07	2.11E-06
AZD6482	p110β	6.65E-06	1.7E-05	1.57E-05	1.25E-05

Sup Figure 1

Characterizing the phenotype of the GDC0032-aquired-resistant cell lines

A. The sensitivity of various ovarian cancer cell lines to BYL719 (arbitrary units) in relation with their PIK3CA state (mutated –red , CNV – orange and wild type – white).
B. Table summarizing the IC:50 (M) of different p110 inhibitors for IGROV1 and OAW42, sensitive (black) and resistant (red) cells.

A

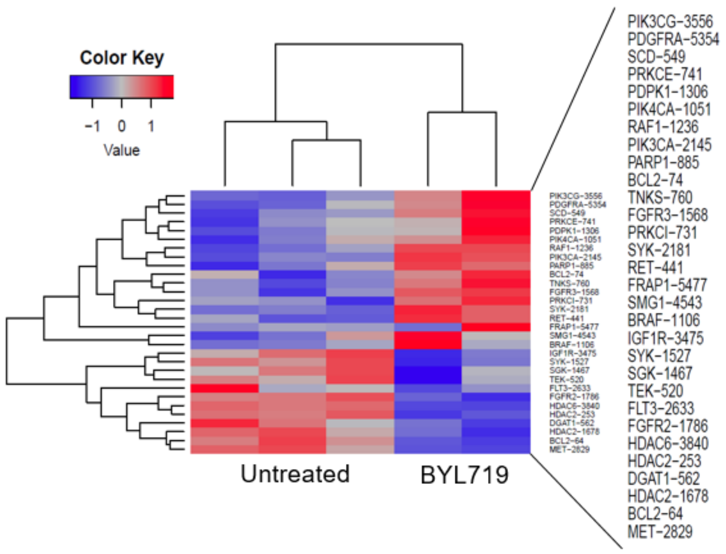

B

Drug Name	Protein
MK2206	AKTi
PHA665752	METi
LDE225	SMOi
17-AAG	HSP90
LEE011	CDK4/6i
LY2835219	CDK4/6i
LBH589	HDACi
AZD6738	ATRi
AEW541	IGF1Ri
AZD6482	P110bi
ABT737	BCL2i
Navitoclax	BCL2i

C

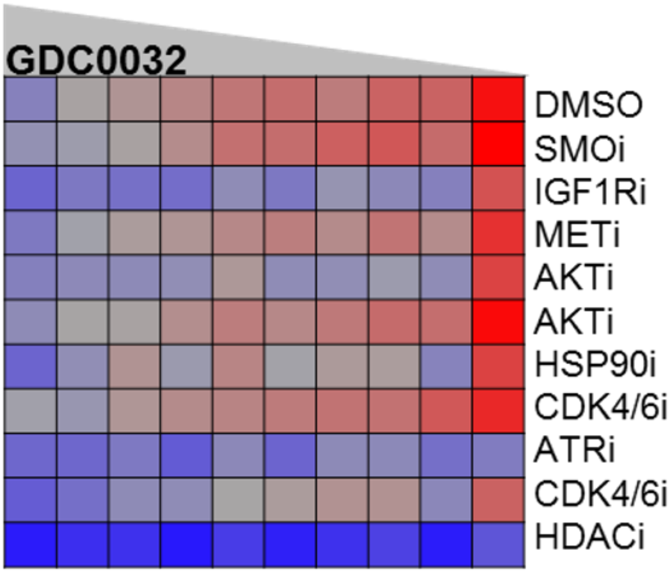

D

Cell line	Synergy score GDC0032+ AEW541	Synergy score BYL719 + AEW541	Synergy score GDC0032+ PHA
IGROV1 Sensitive	1.26	6.42	0.46
IGROV1 Resistant	1.68	2.43	0.31
OAW42 Sensitive	1.55	1.19	0.21
OAW42 Resistant	1.71	1.82	0.71

E

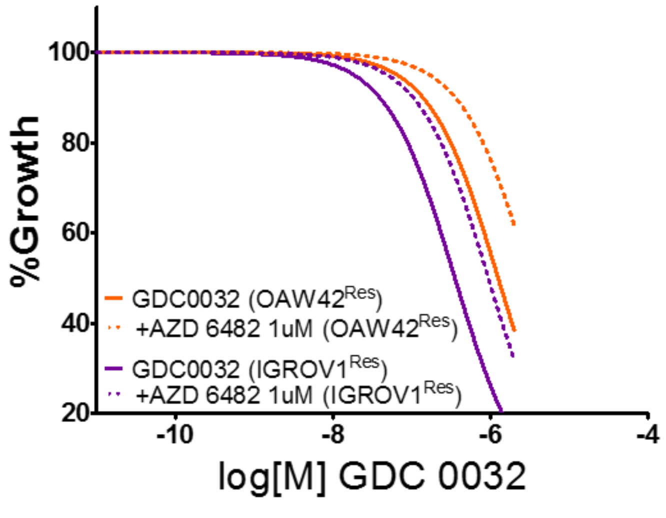

Sup Figure 2

Drug combinations

A. Heatmap across all samples visualizing the top 30 differentially expressed shRNAs for the comparison between the cells treated with BYL719 and the control cells.
B. Table containing the list of inhibitors used and their targets.
C. A drug matrix showing the sensitivity of $OAW42^{Res}$ cells to multiple inhibitors, combined with increasing concentrations of GDC0032.
D. Synergy score of the GDC0032/AEW541, BYL719/AEW541 and GDC0032/PHA665752 combinations, calculated as described in material and methods.
E. GDC0032 IC:50 values of $IGROV1^{Res}$ and $OAW42^{Res}$, with and without the addition of AZD6482.

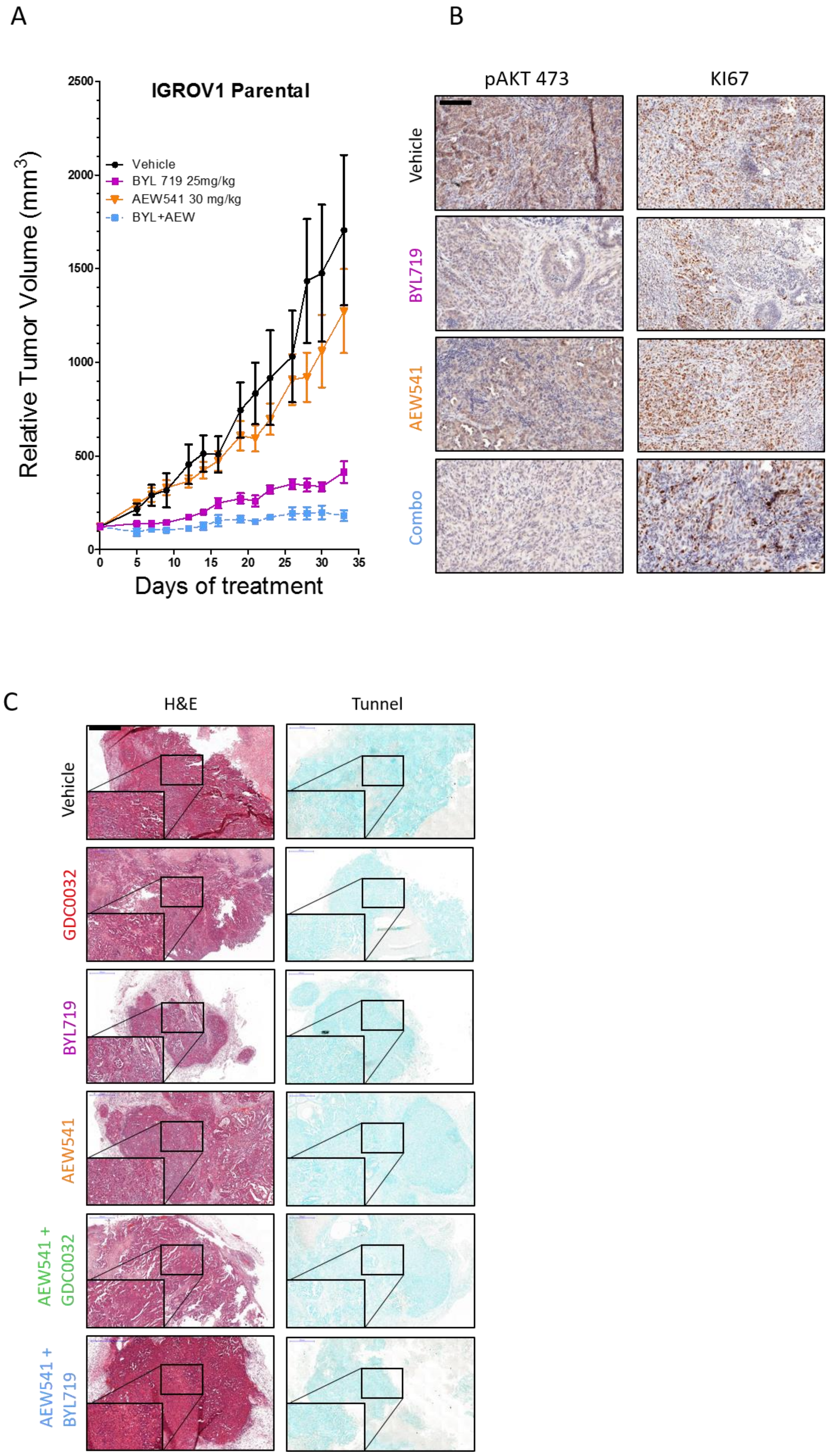
A
B
IGROV1 Parental
Relative Tumor Volume (mm3)
Days of treatment
Vehicle
BYL 719 25mg/kg
AEW541 30 mg/kg
BYL+AEW
0
500
1000
1500
2000
2500
0
5
10
15
20
25
30
35
pAKT 473
KI67
Vehicle
BYL719
AEW541
Combo
C
H&E
Tunnel
Vehicle
GDC0032
BYL719
AEW541
AEW541 +
GDC0032
AEW541 +
BYL719

Sup Figure 3

The combination of BYL719 and AEW541 enhances tumor growth arrest

A. Four arms of tumor bearing mice were treated daily as indicated. Tumor dimensions were measured every two days using a caliper and the relative tumor volume was calculated using the formula: V = (L x W x W)/2, where V is tumor volume, W is tumor width and L is tumor length.
B. KI67 and pAKT473 IHC staining of tumors from the four arms as indicated (Scale bar- 100um).
C. H&E and TUNEL staining of all tumors from the six arms as indicated (Scale bar- 100um).

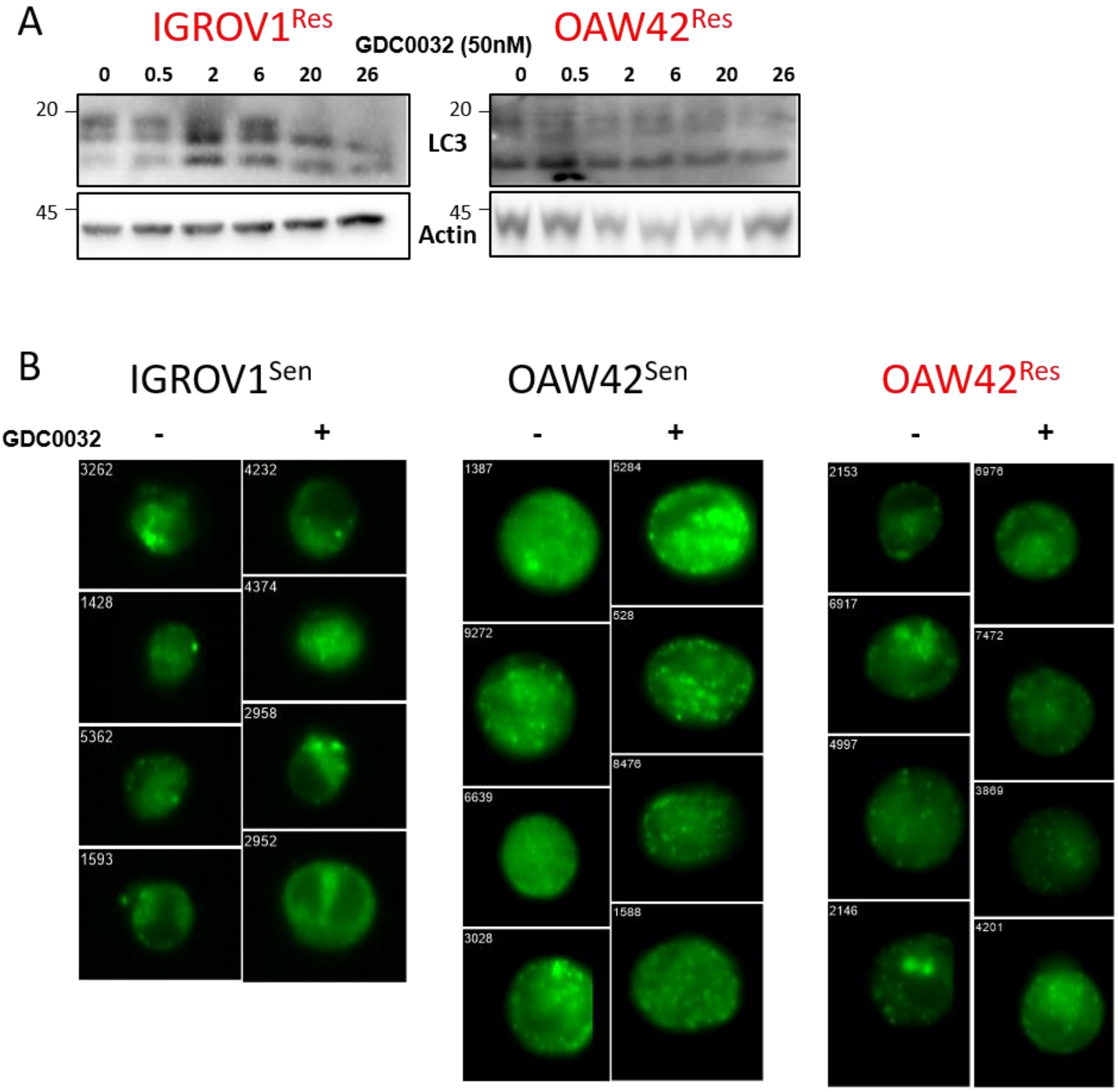
A
IGROV1Res
GDC0032 (50nM)
OAW42Res
0 0.5 2 6 20 26
0 0.5 2 6 20 26
20
LC3
20
45
Actin
45
B
IGROV1Sen
OAW42Sen
OAW42Res
GDC0032
-
+
-
+
-
+
3262
4232
1428
4374
5362
2958
1593
2952
1387
5284
9272
528
6639
8476
3028
1588
2153
6976
6917
7472
4997
3869
2146
4201

Sup Figure 4

IGF1R determines autophagy in OAW42 cells

A. The changes in LC3B levels in both OAW42^{Res} and IGROV1^{Res} were analyzed by whole cell lysate western blot analysis in a time series (hours) of GDC0032 50nM treatment.
B. punctate structures were stained using the Cyto-id kit and average punctate structure per nucleus calculated using the ImageStream count feature. The pictures indicate the positive populations chosen for the count feature.
